# Usability and acceptability of a novel TB infection diagnostic test among key populations in Mexicali

**DOI:** 10.1371/journal.pgph.0005042

**Published:** 2025-08-25

**Authors:** Amanda Brumwell, Rosa Herrera, Kevin Contreras, Mildred Lee, Eduardo Becerra, Julia Estrada-Guzmán, Thomas Nicholson, Rene Machado Contreras, Meredith Brooks

**Affiliations:** 1 Department of Global Health, University of Washington School of Public Health, Seattle, Washington, United States of America; 2 Advance Access & Delivery, Inc, Durham, North Carolina, United States of America; 3 Mexicali Health Jurisdiction Tuberculosis Program, Mexicali, Baja California, Mexico; 4 Universidad Autónoma de Durango, Campus Mexicali, Mexicali, Baja California, Mexico; 5 Universidad Autónoma de Baja California, Mexicali, Baja California, Mexico; 6 Department of Global Health, Boston University School of Public Health, Boston, Massachusetts, United States of America; Pontificia Universidad Católica de Chile: Pontificia Universidad Catolica de Chile, CHILE

## Abstract

Tuberculin skin test (TST) remains the standard-of-care test for TB infection in many high TB-burden settings. Despite existing diagnostics overcoming challenges associated with TST implementation, there has been poor uptake programmatically. We conducted formative research into patients’ and providers’ perceptions of acceptability and usability of a novel IGRA test, called QIAreach QuantiFERON TB (or, QIAreach), compared to TST in a programmatic setting in Mexicali. Programmatic outreach to screen for TB disease and infection was conducted in Mexicali (December 2020-July 2021). A 5-point Likert scale survey was administered to two groups at high risk of TB infection—people who use drugs (PWUD) and household contacts (HHC) of TB patients—and who received testing via TST and QIAreach. This survey evaluated patients’ comparative preferences for the two tests. Additionally, a modified system usability scale was administered to TB program staff involved in the administration and processing of TST and IGRA tests pre- and post-QIAreach implementation to measure the tests’ perceived usability. Of 201 patients, 103 (51.2%) were PWUD and 98 (48.8%) were HHCs. The acceptability survey found that the blood draw for QIAreach was preferred to, and more trustworthy than, the injection for TST. Also, only requiring one visit with providers for QIAreach was preferred among HHCs, but comparatively less preferable for PWUD. In-person delivery of test results was preferable and more trustworthy. The majority preferred QIAreach over TST; though this was higher among PWUD. Nine staff completed the usability survey. Perceived usability before implementing the IGRA remained nearly constant from the pre-implementation timepoint (SUS Score: 52.5, IQR: 45–65) to the post-implementation timepoint (50, IQR: 45-52.5, p = 0.31). IGRA was more acceptable to patients, and perceived usability was mixed among staff. Patient and provider preferences must be considered when integrating novel IGRA tests into settings where TST is routinely used.

## Introduction

Globally, 1.8 billion people are infected with tuberculosis (TB); an estimated 10% progress to TB throughout their lifetime, with the majority doing so withing two years [1]. Taking TB preventive treatment (TPT) can reduce the risk of progression from TB infection to disease by 60–90% [2,3]. Finding people with TB infection who are at risk of disease progression and initiating them on TPT is essential to prevent further transmission and ultimately reduce TB incidence globally. Reducing TB transmission is a crucial means to achieve the of the WHO End TB strategy’s target of reducing TB incidence globally by 80% by 2030 [4].

Individuals who are household contacts of people with TB disease and individuals with behavioral risk factors like drug use are at high risk of TB infection [5]. Thus, appropriate testing of these key and vulnerable populations at high risk for TB infection is essential to rapidly initiate TPT. Several TB infection tests are recommended by the WHO for identifying TB infection and used globally, including Tuberculin Skin Tests (TST) and Interferon Gamma Release Assays (IGRA) [6]. TST is often the standard of care for diagnosing TB infection in many high TB-burden countries globally; however, there are many challenges introduced by TST, including global stockouts of tuberculin [7], the requirement for individuals to return to a health facility after 48–72 hours for their TST induration to be interpreted, and heterogeneity in sensitivity and specificity [8,9]. IGRAs are more sensitive than TSTs (between 90–98% [8,9]) and overcome many of the operational challenges of TST, including only requiring a single visit and no cross-reactivity with BCG vaccination [10]. However, IGRA requires a functional laboratory for testing blood samples using enzyme-linked immunosorbent assays (ELISA).

QIAreach QuantiFERON TB (hereinafter referred to as QIAreach) is a novel IGRA test that requires 1mL of blood and no ELISA equipment for analysis [11]. This presents operational advantages over traditional IGRA tests, which require specialized laboratory equipment and facilities, sophisticated supply chains, a larger blood draw, and trained laboratory staff. Traditional IGRA tests require a comparatively complex process for analyzing results, which can lead to delays in identifying TB infection. In comparison, the QIAreach requires less blood for its sampling and does not require sophisticated laboratory infrastructure for analysis, with the testing primarily conducted using an incubator, standard pipettes, and the testing console, which may be operated in non-laboratory settings.

Prior research has demonstrated that IGRA are more cost effective than TST after accounting for investment in laboratory infrastructure, lost opportunity costs incurred by healthcare staff and people undergoing TB infection testing, and ongoing TB transmission due to cases missed by TST’s lower specificity and sensitivity [12]. Further, in an analysis assessing the concordance of QIAreach and TST, we found that QIAreach is an effective tool for identifying TB infection in high-burden populations where TST is the standard of care [13–15]. QIAreach has strong diagnostic performance compared to other IGRA diagnostic tools, particularly in moderate-to-high risk populations [16], although recent studies have suggested heterogeneous concordance and an increased risk of false-positive results with QIAreach compared to QuantiFERON-TB Gold Plus, another QIAGEN diagnostic system, among members of the general population [17,18].

Despite evidence of economic and effectiveness advantages of IGRA over TST, the successful integration of IGRA into existing programmatic care also depends on patient-level acceptability and health facility- and laboratory staff-level usability of the tests. Thus, we assess the acceptability among patients and usability among TB program staff for QIAreach compared to TST in a programmatic setting in Mexicali, Mexico.

## Methods

### Ethics statement

IRB approval for this study was issued by the General Hospital Ethics Committee of Mexicali, (02–01-HGMXL/UABC/ISESALUD/QIAGEN/2020-11-20-279). Written informed consent was provided prior to enrollment. All data was managed under the privacy policies of the health care services of the Mexicali Jurisdiction.

### Setting and program description

Mexicali, Baja California is a city of roughly one million people and with a tuberculosis prevalence of approximately 52 per 100,000, one of the highest rates of TB in North America. Beginning in 2019, the Mexicali health jurisdiction has implemented an intensified patient-finding and elimination strategy that expands on the Mexican national guidelines to prioritize patient-finding in key populations, expand testing for TB infection, and improve TPT prescription for people belonging to high-risk populations and for whom TB disease has been ruled out. As described elsewhere, TST is the standard of care for diagnosing TB infection, and six months of isoniazid is routinely used for TPT when active disease is ruled out.

Between December 2020 and January 2022, the Mexicali TB program piloted the use of QIAreach as a part of programmatic, active patient-finding. The performance of QIAreach was compared against that of TST; this work, the program’s methods for gathering informed consent, conducting TB evaluations, and its findings have been described elsewhere [13–15]. QIAreach and TST were administered for programmatic TB infection screening among two groups at high risk of TB: people who use drugs (PWUD), and household contacts (HHC) of people diagnosed with TB disease. In the general population, cross-border migration, particularly among binational individuals, complicate TB programs’ ability to trace contacts of people with TB and ensure that treatment is completed [19,20]. A high burden of co-morbidities, such as obesity and type II diabetes, as well as low income and TB-related stigma, together limit access to medical services and complicate TB diagnosis and treatment [20,21]. PWUD were residents of a rehabilitation facility treating addiction to alcohol, methamphetamine, cocaine, and/or heroine. As residents of this facility, PWUD often have restricted access to medical care, and addiction in this population is associated with increased TB incidence and reduced likelihood of TB treatment completion [19,20]. This rehabilitation facility received outreach services from the TB program in December 8, 2020. HHC were contacts of people diagnosed with TB disease in July 2021. They were identified from the list of contacts enumerated in the program’s TB register.

### Acceptability survey

All individuals who received both tests were administered an 8-question, 5-point Likert scale survey that evaluated their preferences and trust for the TST testing process compared to that of QIAreach. The survey’s domains were determined through a process mapping exercise that identified key differences in the TST and QIAreach testing processes from the patient’s perspective: specimen collection, the number of patient-provider interactions required, and the means through which results are delivered. From these domains, the study team defined eight questions related to preferences and perceived trustworthiness. The questions were translated into Spanish by programmatic partners, and they were refined for comprehension before being implemented. The final questions were related to the following topics: preference for blood draw or injection, trustworthiness of blood draw versus injection, preference for one or two visits with provider, preference for learning test results remotely or in person, trustworthiness of learning results virtually versus in person, preference for QIAreach or TST testing process, trustworthiness of QIAreach or TST, recommendation for QIAreach or TST. Responses to each question were on a 5-point Likert scale to evaluate comparative preferences for QIAreach and TST; options were ‘Strongly prefer QIAreach’, ‘Somewhat prefer QIAreach’, ‘No preference for QIAreach or TST’, ‘Somewhat prefer TST’, and ‘Strongly prefer TST’ [S1 Data].

### Usability survey

A modified system usability scale to assess pre- and post-use perceptions of usability of the two tests. The system usability scale (SUS) [22] was selected for its brevity, widespread validation in usability research [23], and because it has been validated in Spanish [24]. This scale includes ten questions that compares one system or tool to another using a 5-point Likert ranging from strong preference for TST to strong preference for IGRA. The questions include: which test the respondent would like to use frequently, which test is more complex, which test is easier to use, for which test the respondent feels they would require the support of a technical person for use, which test had more integrated functions, which test had more inconsistency, which test would be faster to learn how to use, which test would be more cumbersome to use, which test a participant would be more confident using, and which test would require the respondent to learn more before it could be used. Each of these questions was evaluated using a Likert scale response using the following points: TST strongly satisfies this statement, TST somewhat satisfies this statement, QIAreach and TST equally satisfy this statement, QIAreach somewhat satisfies this statement, and QIAreach strongly satisfies this statement.

This survey was administered to staff responsible for administering TST and QIAreach at two time points. The first time point was after a training for how to use QIAreach and a training to review the correct use of TST in October 2020. The second time point was after the program team had administered QIAreach and TST to the group of PWUD in January 2021 [S1 Data].

### Analysis

The results of the acceptability survey were calculated using summary statistics. For the usability survey, we calculated the median and IQR SUS scores, which were assessed according to the scale’s instructions within a scoring range of 0–100 [22]. A low score indicates low overall usability for the new testing system compared to the standard of care, and a high score indicates high overall usability for the new testing system compared to the standard of care. Usability survey scores from before and after diagnostic test administration were compared using the Wilcoxon signed rank test to account for multiple, non-normally distributed observations across the same individuals using R Studio version 2022.02.1 + 461.pro1 “Prairie Trillium”.

### Inclusivity in global research

Additional information regarding the ethical, cultural, and scientific considerations specific to inclusivity in global research is included in the Supporting Information (S1 Checklist).

## Results

In total, 201 patients completed the acceptability survey, including 103 PWUD and 98 household contacts (Table 1).

**Table 1:** Demographics of acceptability survey participants.

	PWUD (n = 103)	HHC (n = 98)	TOTAL (n = 201)
Median age (IQR)	34 (27-42)	27 (8.75-45)	31 (22-43)
Age group			
* 0-19*	1 (1.0%)	35 (35.7%)	36 (17.9%)
* 20-39*	63 (61.2%)	35 (35.5)	98 (48.8%)
* 40-59*	39 (37.9%)	21 (21.4%)	60 (30.0%)
* 60+*	0 (0)	7 (7.1%)	7 (3.5%)
Sex: Male	103 (100.00%)*	51 (52.0%)	154 (76.6%)
BCG Vaccinated	86 (83.5%)	94 (95.9%)	180 (89.6%)
HIV-positive	19 (18.45%)	1 (1.02%)	20 (10.0%)
Close contact of TB patient	26 (25.24%)	98 (100.00%)	124 (61.7%)

*All PWUD male due to the nature of the rehabilitation facility in which they resided at the time of the study enrollment.

**Fig 1:**
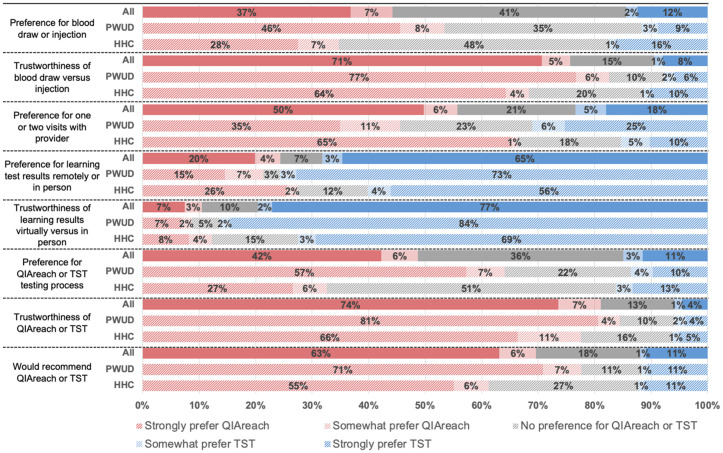
Preferences for QIAreach compared to TST in aggregate and by risk group.

Participant preferences are shown in Fig 1. 44% overall preferred the blood draw for QIAreach compared to the TST injection, and 41% had no preference. By risk group, PWUD had a higher preference for a blood draw compared to the TST injection (54%) than did HHC (35%). HHC had a higher percentage of respondents indicating no preference for a blood draw or injection (48% compared to 35% PWUD).

A blood draw was considered more trustworthy compared to an injection by 76% of respondents. 83% of PWUD felt a blood draw was more trustworthy than an injection compared to 68%. Most participants (56%) preferred one visit with a provider, which is possible through QIAreach, compared to two visits as required by TST. This includes 44% of PWUD and 66% of HHC who preferred one visit to two visits. Notably, 31% of PWUD preferred two visits with a provider, compared to 24% HHC. Learning test results in person, which is possible for TST, was considered more preferable by 68% of participants and more trustworthy by 79% of participants compared to learning results online or over phone.

The majority of participants preferred QIAreach to TST (48%) and felt QIAreach was the more trustworthy option (81%), although when disaggregating participants by sub-group, 51% of HHC did not feel a preference towards either test, compared to 22% of PWUD. 69% of participants said they would recommend QIAreach and 12% said they would recommend TST; 11% of PWUD and 27% of HHC indicated that they were impartial to recommending either test.

**Fig 2:**
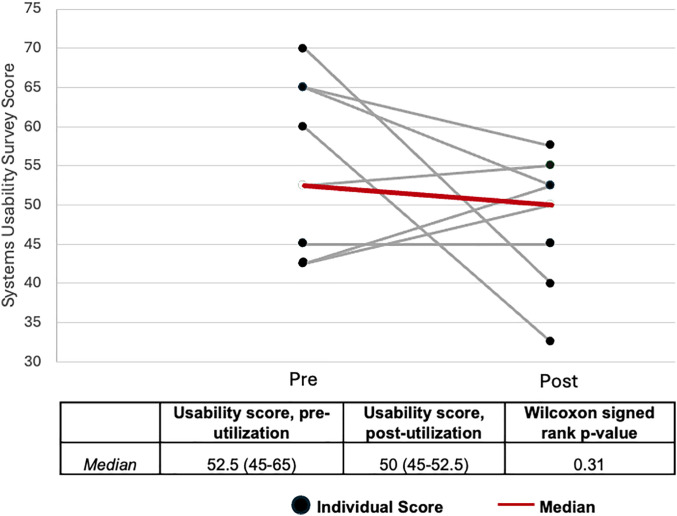
Providers’ usability scores comparing QIAreach to TST, pre- and post-utilization of QIAreach.

Nine TB program staff completed the usability survey (Table 2). Four (44.4%) of the staff were female, 6 (66.7%) worked as health promoters, 2 (22.2%) worked as physicians, and one worked as a directly-observed therapy nurse. Individual responses pre- and post-implementation are presented in Fig 2. Perceived usability of QIAreach increased for three participants, decreased for four participants, and remained the same for two participants from the pre- to post-implementation time point. The median usability score after training and before test implementation was 52.5 (IQR 45–65), where a higher score indicates higher perceived usability than the comparator [22]. After test implementation, the usability score was 50 (IQR 45-52.5). There was not a statistically significant difference between the pre and post usability scores (p = 0.31).

**Table 2:** Demographics of usability survey participants.

	N (%)
Total number surveyed	9
Age Group	
* 20-29*	7 (77.8%)
* 30-39*	0 (0)
* 40-49*	2 (22.2%)
Gender: Female	4 (44.4%)
Role	
* Health promotor*	6 (66.7%)
* Physician*	2 (22.2%)
* Directly Observed Therapy (DOT) Nurse*	1 (11.1%)

## Discussion

The novel IGRA, QIAreach, was found to be more acceptable among patients compared to TST, although there is important heterogeneity between key populations. The QIAreach QFT testing process may have advantages over the standard of care, TST. Many patients preferred phlebotomy to the tuberculin injection, although a notable minority (35.0%) did not have strong preference for either process. Patients largely felt that the phlebotomy was more trustworthy and that they would recommend the QIAreach test. However, patients felt less strongly about the testing overall. This may be related in part to their preference for components of the TST testing process including the necessity for two visits with care providers and communication of their test results in person. In contrast, the QIAreach does not require a second visit, and as a result the QIAreach enables results to be communicated virtually.

Staff members involved in TST and QIAreach administration reported heterogeneous usability scores comparing QIAreach to TST. Raw scores indicated that perceived usability of QIAreach remained nearly constant from pre-IGRA usage (score of 52.5) to post-IGRA usage (score of 50). It is interesting to note that this heterogeneity is the result of individual-level changes in usability pre- and post-implementation, rather than heterogeneous results that remained constant after using QIAreach. The differences pre- and post- in two SUS questions may indicate that the staff’s level of comfort using QIAreach did not change substantially between the two timepoints. Further research may be warranted to assess why users’ opinions of QIAreach usability changed in both the positive and negative direction. It may also indicate the importance of training and knowledge for use of the QIAreach among staff members. First, staff indicated a stronger need for the support of a technical person to use QIAreach in the post-survey. Second, staff also felt more strongly about the learnability of QIAreach after using QIAreach.

Patient- and provider-level preferences are important determinants to successful diagnosis, treatment completion, and likelihood of loss to follow-up, and yet the preferences of the people most affected by TB have been understudied [25,26]. Our findings have important implications for initiatives seeking to integrate novel TB infection diagnostics into their existing programs and for improving patient-centered care delivery to vulnerable patients. Patient-centered models of TB diagnosis and treatment are a key priority for the WHO’s End TB strategy as a means to improve the quality and effectiveness of care, by addressing barriers to care, improving acceptability of services, and promoting the adherence and engagement of people with TB [27]. Importantly, we believe that our findings underscore one size does not fit all in TB program design and care delivery [28], and this study provides much-needed evidence towards operationalizing differentiated models of care that are suited to the needs and preferences of two different groups, HHC and PWUD [29].

First, more opportunities to interact with care providers may improve the diagnostic experience for vulnerable patients like PWUD, who likely experience restricted access to medical attention [30,31]. This is particularly important in this setting, where the PWUD participants were residing in a rehabilitation center which they were unable to leave. Although the largest proportion of respondents preferred one visit from a care provider, nearly a third of respondents preferred or strongly preferred two visits. This must be balanced against patients’ preferences for receiving test results quickly or same-day, which was not measured in this study but has been reported elsewhere [26,32]. Reasons for the preferences identified in this study should be further explored in order to understand acceptable strategies for care delivery for high TB burden populations. However, we expect their preference for multiple visits may be related to a few contextual factors. PWUD in Mexico tend to face more severe financial barriers to care, which may increase the value placed on clinical interactions [33]. Further, a combination of stigma related to drug use and to a TB diagnosis may cause PWUD to feel particularly isolated from the health care system, again increasing the value placed in non-stigmatizing, patient-centered services [34].

Second, people with TB’s perceived trustworthiness of testing processes, including the delivery of testing results, should inform improvements to program activities. Participants indicated that a blood draw, associated with the QIAreach, and receiving results in person, which is facilitated by but not exclusive to the TST, are both considered more trustworthy compared to the alternatives. Faith in diagnostic testing processes is an important determinant for the timeliness of TB diagnosis and care [35]. These findings must be tempered against our observation that some participants felt no preference for QIAreach’s blood draw over the TST injection, and similarly no preference for one testing process over the other. These neutral responses may be due to the fact that both processes are relatively invasive, and we may expect to see relative preference for a testing process requiring a less-invasive sample, such as urine or saliva [36,37]. Although the QIAreach testing process was more trustworthy overall, programs may consider in-person communication of testing results in the delivery of IGRA test results. While there are currently no IGRA test options that provide immediate results, which would facilitate in-person communication, programs may consider models that balance these two needs, such as home-based results delivery through community health workers, or rapid linkage to facility-based care once results are available.

Third, comprehensive training is essential for integrating a novel diagnostic test into an existing community-based, care delivery program. Technical support was deemed important for using the QIAreach, but staff felt that most people could learn to use the test quickly. Considering that QIAreach is intended for use in settings where IGRA diagnostics may not be routinely used, training for phlebotomy, sample storage and preparation, analysis, and data management is an important step towards ensuring staff confidence in the novel testing process, reducing the likelihood of care delays or poor quality of care, and promoting the sustainability of the program beyond initial integration efforts [38]. Notably, in a Zambian setting where IGRA technologies are routinely used, QIAreach not only offered operational advantages compared to other IGRA technologies currently in use, but users with prior experience of IGRA technologies reported higher QIAreach usability compared to those without this experience [39]. This may highlight the opportunity to improve training by increasing providers’ exposure not only to new technologies but to novel training modalities, using methods such as audit and feedback loops, which offer iterative guidance and troubleshooting, refresher trainings that focus on behavior change [40], or co-design of training packages to incorporate the design preferences of providers and test users at the onset of technology adoption [41,42].

Of note, the QIAreach diagnostic test is not currently being manufactured nor available for purchase. However, the findings of this study demonstrate the preferences of patients and comparative usability of aspects of QIAreach that are shared more generally with the process of testing using IGRA tests. Among healthcare workers who would use IGRA in their routine care of people with presumed TB infection, we may still conclude that an IGRA test may be usable. And, among people with presumed TB infection, we may still conclude that a testing process involving blood collection, rapid communication of in-person results, and fewer in clinical visits would be acceptable. Should a future TB infection test share these features, particularly a TB infection test that is near-patient and easy to use, it would be likely that this test would be accepted and well-adopted. Moreover, given the differential preferences between patient groups, it is recommended that providers engage with their patients to determine their needs and preferences in order to select the TB infection test that is best-suited to their circumstances.

This study has a number of limitations and strengths. Among its limitations is the small sample size of individuals completing the usability survey, which may limit the generalizability of our findings. However, a variety of staff working in different patient-facing roles completed the survey, which may mitigate limitations of the relatively small sample size. This study was also not designed with statistical power, and therefore it is difficult to determine whether our findings indicate there was truly no change in perceived usability, or that we were simply unable to detect a change. A larger sample size may have improved our ability to generalize our findings, increase the precision of our estimated change in usability post-training, and reduce the possibility of type II error – that is, incorrectly concluding that was no treatment effect. Had we observed a statistically significant difference, particularly in a larger population, we would have been able to conclude with greater certainty that the novel diagnostic was more usable than TST following training and real-world experience. However, our findings from this formative research will be informative for statistical powering of future usability studies of similar diagnostic tools. Another limitation includes the timing of this study, which coincided with the covid-19 pandemic, which precipitated substantial changes in health care services that may have altered individuals’ perceptions of health and care. The effects of the pandemic including the public response to outbreaks and public health control measures may have affected participants’ attitudes towards tuberculosis infection testing, although these effects have not been well studied in this setting. Health care utilization was diminished during the covid-19 pandemic, which may reflect limited access to care; we hypothesize that this may cause individuals to place greater value in clinical services as well as decentralized care provision [43]. It is likely that providers were under greater stress during this time [44], which may have affected their ability to retain training to use QIAreach.

Among this study’s strengths are its use of a robust and validated usability score that has been applied in a variety of settings. Also, this study developed and applied an easy-to-use survey for measuring acceptability that accounts for various aspects of the testing process from the perspective of someone with presumed TB. This study also surveyed two different high TB-risk populations, including one, PWUD, who are particularly vulnerable and whose preferences for TB care delivery are understudied. Given the high burden of TB infection among PWUD [13,45], we anticipate that our findings may be informative for future efforts to tailor TB programs to the needs of this population. We suggest that future research utilize mixed-methods approaches to assess both qualitatively and quantitatively the strengths and limitations of QIAreach compared to TST, especially from the providers’ perspectives. This would be informative for developing more effective training and integration plans for novel IGRA and near-patient technologies. Future qualitative initiatives may engage with a broad range of stakeholders to co-design TB infection testing models maximize the advantages of diagnostics’ design and streamline care delivery. They may also investigate patient care pathways and explore barriers and facilitators to diagnostic delivery from the patient and provide perspective. They may also investigate providers’ preferences for TB testing to promote diagnostic usability.

In conclusion, we found that QIAreach, a novel IGRA, was considered acceptable to patients in a Mexican setting where TST is the standard of care. We noted differences in the responses of HHC and PWUD, and the preferences that these responses suggest should be considered when integrating new IGRA technologies into TB elimination strategies. This is particularly true for IGRA technologies and newer diagnostics that may be delivered closer to community settings, with fewer barriers to implementation such as less specialized equipment and less invasive sampling procedures. We also found that QIAreach is considered to be moderately usable by TB program staff, and we suspect that additional training may aid in the usage of this diagnostic tool. We recommend further research to understand and evaluate effective strategies to implement novel IGRA technologies like IGRA into high-burden settings where IGRA is not routinely used.

## Supporting information

S1 Data Usability and Acceptability Datasets and Codebooks. (XLSX)

S1 Checklist Inclusivity in Global Research Questionnaire. (DOCX)
